# Assessing Genetic Diversity for a Pre-Breeding Program in *Piaractus mesopotamicus* by SNPs and SSRs

**DOI:** 10.3390/genes10090668

**Published:** 2019-08-31

**Authors:** Vito Antonio Mastrochirico-Filho, Felipe del Pazo, Milene Elissa Hata, Gabriela Vanina Villanova, Fausto Foresti, Manuel Vera, Paulino Martínez, Fábio Porto-Foresti, Diogo Teruo Hashimoto

**Affiliations:** 1 Aquaculture Center of Unesp, São Paulo State University (Unesp), Jaboticabal, SP 14884-900, Brazil; 2 Laboratorio Mixto de Biotecnología Acuática - Universidad Nacional de Rosario, Facultad de Ciencias Bioquímicas y Farmacéuticas - Ministerio de Ciencia, Tecnología e Innovación productiva de Santa Fe. Centro Científico y Tecnológico Acuario del Río Paraná, Rosario, Santa Fe 2000, Argentina; 3 Consejo Nacional de Investigaciones Científicas y Técnicas (CONICET), Rosario, Santa Fe 2000, Argentina; 4 Institute of Biosciences, São Paulo State University (Unesp), Botucatu, SP 18618-970, Brazil; 5 Facultad de Veterinaria, Universidad de Santiago de Compostela (USC), ES27002 Lugo, Spain; 6 Instituto de Acuicultura, Universidad de Santiago de Compostela (USC), 15705 Santiago de Compostela, Spain; 7 São Paulo State University (Unesp), School of Sciences, Bauru, SP 17033-360, Brazil

**Keywords:** aquaculture, genetic variability, inbreeding, breeding programs

## Abstract

The pacu (*Piaractus mesopotamicus*) is a Neotropical fish with remarkable productive performance for aquaculture. Knowledge of genetic resources in Neotropical fish is essential for their applications in breeding programs. The aim of this study was to characterize the genetic diversity of seven farmed populations of pacu which will constitute the basis for a broodstock foundation for coming breeding programs in Brazil. Analysis of one wild population (Paraná River) was used as a reference to compare genetic parameters in the farmed populations. The analyses were performed using 32 single-nucleotide polymorphisms (SNP) and 8 simple sequence repeat (SSR) markers. No significant differences in genetic diversity between populations estimated through the number of alleles and allelic richness, observed heterozygosity, expected heterozygosity, and minimum allele frequency were detected (*p* > 0.05). Low genetic diversity was observed in all farmed stocks and the wild population. Moreover, we detected low genetic structure when comparing farmed and wild populations for SNPs (*F*_ST_ = 0.07; K = 3) and SSRs (*F*_ST_ = 0.08; K = 2). Analysis of molecular variance (AMOVA) demonstrated that genetic variation was mostly within populations. Kinship analysis showed that most fish farms included related individuals at a proportion of at least 25%. Our results suggest that the basal broodstock for pacu breeding programs should be founded with individuals from different fish farms for higher genetic diversity and to avoid inbreeding risks.

## 1. Introduction

The pacu (*Piaractus mesopotamicus*) is a Characiform fish with a wide natural distribution throughout La Plata basin which covers an area over five South American countries: Brazil, Uruguay, Bolivia, Paraguay, and Argentina. Wild populations of pacu are threatened by overfishing since this species is considered to have high commercial value, with large-scale catches occurring by industrial and recreational fisheries [1]. According to the latest official statistics on industrial fisheries in Brazil, wild catches of pacu represent 5% (11 thousand tons) of the total inland capture (243.8 thousand tons) [2], classifying it as a critically endangered species in Sao Paulo State in Brazil (Decree 56,031). In relation to aquaculture production, pacu is one of the most cultivated fish species in South America. In Brazil, pacu farming already represents the second largest harvest from fish production (about 21 thousand tons) among native species [3]. According to its productive characteristics, it is an omnivorous fish considered a gainful species for aquaculture due to its rapid growth and low-cost feeding habits [4]. Due to its productive viability, its production in aquaculture has increased even in other parts of the world, such as China, Myanmar, Thailand, and Vietnam [5,6].

Despite breeding programs being fundamental for the sustainable development of aquaculture, less than 10% of aquaculture production in the world is based on genetically improved stocks, limited to only a few species [7], and there is no production of native species from Latin America resulting from genetic selection. For successful breeding strategies, suitable pre-breeding programs have been fundamental to providing advantageous and enduring genetic gains for fish production [8]. Therefore, evaluations of genetic variability and genetic relationships in the base population are essential for effective control over generations, which reduces problems related to loss of genetic potential for selective breeding in farmed stocks [9].

Mass selection without appropriate genetic supervision usually intensifies the processes of genetic drift, determining increased inbreeding and a reduction in genetic variability. Consequently, negative impacts such as reduced growth rates, low survival, morphological deformities, and susceptibility to disease have been reported [10,11,12]. Thus, the evaluation of genetic diversity and population structure by molecular markers to assist directed mating is important for successful management in selective breeding programs [13,14].

In pacu, genetic diversity studies have been limited to evaluating wild populations using the mitochondrial DNA control region [15] and microsatellite markers (or simple sequence repeats, SSRs) [16,17]. Both types of studies have characterized wild populations of pacu as a panmictic stock with high gene flow among rivers.

In this study, seven farmed stocks and one wild population of *P. mesopotamicus* were examined by using 32 single-nucleotide polymorphisms (SNPs) and 8 SSRs to assess the genetic diversity, structure, and kinship as a support to designing a suitable pre-breeding program for this species.

## 2. Materials and Methods

### 2.1. Ethic Statement

This study was conducted in strict accordance with the recommendations of the National Council for Control of Animal Experimentation (CONCEA) (Brazilian Ministry for Science, Technology and Innovation) and was approved by the Animal Use Ethics Committee (CEUA), protocol no. 22.255/15. The present study was performed under authorization no. 33435-1 issued through the Chico Mendes Institute for the Conservation of Biodiversity, Brazilian Ministry for the Environment (ICMBio). Fin clips were collected from each fish under benzocaine anesthesia and all efforts were made to minimize suffering. Fin samples were stored in 95% ethanol at −20 °C.

### 2.2. Experimental Population

Individuals of seven different broodstocks from fish farms in Brazil (São Paulo State) were collected (FF1–FF7) (number of individuals shown in Table 1). The commercial identity and localization of the fish farms were kept confidential. Animals were individually tagged with transponders (passive integrated transponder tags (pit-tags), model full-duplex FDX-B, 134.2 kHz) and kept alive for subsequent management to create the basal broodstock for the genetic breeding program. Fish farms were mostly set up between the 1980s and 1990s. There are no records of selective mating, except for at FF4. Additionally, 34 individuals collected in the Paraná River, Brazil (latitude −21.39, longitude −51.93) and previously used for SNP validation [18] were used as the reference wild population (Table 1). According to previous studies of genetic structure, wild populations of pacu present high levels of genetic similarity, representing a panmictic unit [15,17]. Therefore, we considered that this particular wild population (Paraná River) would represent the panmictic stock of the natural populations, being used as a reference for comparison of the genetic parameters.

### 2.3. SSR and SNP Analysis

DNA extraction was performed using the saline extraction protocol [19]. DNA integrity was evaluated on 1% agarose gel and its purity was assessed using a NanoDrop One spectrophotometer (Thermo Fisher, Madison, USA). The DNA concentration was quantified using the Qubit dsDNA BR Assay kit (Life Technologies, Oregon, USA) and measured in a Qubit 3.0 Fluorometer (Invitrogen, Kuala Lumpur, Malaysia).

SSR genotyping was performed using eight microsatellite markers (Pm1, Pm3, Pm5, Pm11, Pm4, Pm6, Pm9, and Pm13) in two Multiplex PCR reactions according to Posner [20] and previously standardized using fluorescent labeled primers by CONICET Service (Stan CONICET No. 2353, CCT Rosario, Argentina; http://vinculacion.conicet.gov.ar/buscador-de-oferta-tecnologica/?id_ot=2353&tipo=3). Markers were genotyped in a 3730XL DNA analyzer (Applied Biosystems) in Macrogen (Korea). Allele scoring was performed with the Peak Scanner program (Applied Biosystems) using the GeneScan 500 LIZ Size Standard.

SNP genotyping was carried out using 32 markers obtained from the liver transcriptome [18]. Analysis was performed using the MassARRAY platform (Sequenom, San Diego, CA, USA) in CeGen (Spanish Genotyping National Center, Santiago de Compostela node, Spain) as described in Mastrochirico-Filho et al. [18]. SNP data from the wild population (WILD) were previously reported by Mastrochirico-Filho et al. [18].

### 2.4. Statistical Analysis

The presence of null alleles (F_null_) and allelic dropout in microsatellite loci were tested using MICRO-CHECKER 2.2.3 [21]. The number of alleles per locus (N_a_), number of private alleles (N_p_), and observed (H_obs_) and expected (H_exp_) heterozygosity were estimated using CERVUS 3.0 [22]. The minimum allele frequency (MAF), exact tests for deviation from the Hardy–Weinberg equilibrium (HWE) (Markov Chains of 100,000 steps), and linkage disequilibrium (LD) (*p* < 0.05) were performed using GENEPOP 4.0.11 [23]. The allelic richness (A_r_) and inbreeding coefficient (*F*_IS_) were estimated using FSTAT 2.9.3.2 software [24]. Bonferroni correction was applied for multiple tests [25].

The effective population size (N_e_) was estimated by the linkage disequilibrium method in NeESTIMATOR V2.01 [26] and was used for a gross estimation of inbreeding in the analyzed fish farms (∆F = 1/2Ne) [27]. Recent bottleneck events were evaluated by M-ratio testing [28] using ARLEQUIN version 3.5.2.2 [29]. Bottleneck analysis was performed only for SSR data because of the biallelic nature of the SNPs.

To estimate genetic differentiation between the stocks, global and pairwise *F*_ST_ values were calculated using FSTAT version 2.9.3.2 [24]. The significance of these values was estimated with 10,000 permutations.

Levels of admixture among stocks were inferred by estimating the optimum number of population clusters (K) [30] using STRUCTURE version 2.3.4 [31] without prior information about the population. Primarily, we determined the distribution of ΔK, an ad hoc statistic based on the rate of change in the log probability of data between successive K values. The range of clusters (K) was predefined from 1 to 8. The analysis was performed in 80 replicated runs (i.e., 10 replicates for each K value) using 500,000 iterations after a burn-in period of 100,000 runs. The most likely K value to explain the population structure was the modal value of this ΔK [30]. The outputs of STRUCTURE analysis were visualized through the STRUCTURE HARVESTER program [32]. The results of independent STRUCTURE runs were summarized and corrected for the best K using CLUMPP software version 1.1.2 [33].

The partitioning of variation at different hierarchical levels was calculated by analysis of molecular variance (AMOVA) in ARLEQUIN version 3.5.2.2 [29] using 10,000 permutations. Stocks were grouped according to the clusters obtained by STRUCTURE software.

Kinship coefficients (r_xy_) [34] and potential for each locus to exclude a false parent were estimated using both SSR and SNP loci by COANCESTRY v. 1.0.1.8 [35]. Parentage exclusion probabilities of loci were determined when one individual taken at random from the population was excluded as a parent when no parent was known (PE2). This analysis was performed to evaluate with reliability the pairwise relatedness between farmed breeders from each fish farm without any parental information. Threshold values of the kinship coefficient were adopted as lower values (r_xy_ < 0.125) corresponding to unrelated individuals; intermediate values 0.125 ≤ r_xy_ ≤ 0.375 were considered half-siblings; and r_xy_ ˃ 0.375 were considered full siblings [36].

## 3. Results

The parameters of genetic variability for pacu populations determined by 8 SSR and 32 SNP markers are shown in Tables S1 and S2, respectively. The mean values of the population parameters and overall locus *p* values of HWE and *F*_IS_ values are shown in Table 1.

For SSR loci, a total of 44 alleles were detected in the analyzed populations. The average number of alleles per locus was 3.84 ± 1.16, with numbers ranging between 2 and 7. Allelic richness ranged from 2.000 to 6.140, with average population values ranging between 3.331 ± 0.868 (FF1) and 3.975 ± 1.189 (FF7). Private alleles were detected in individuals belonging to FF2, FF4, FF7, and WILD.

Monomorphic SNPs were found more frequently in FF2 (six monomorphic SNPs) and FF4 (four monomorphic SNPs). By contrast, FF1, WILD, FF6, and FF7 showed only polymorphic SNPs and the highest MAF average values (0.285 ± 0.133, 0.275 ± 0.132, 0.273 ± 0.127, and 0.270 ± 0.144, respectively). On the other hand, FF3 (0.269 ± 0.138) was the fish farms with the lowest MAF average values.

The average H_obs_ was 0.502 ± 0.219 for SSRs and 0.391 ± 0.173 for SNPs. When the average H_exp_ was estimated, 0.525 ± 0.170 was found for SSRs and 0.379 ± 0.133 for SNPs. The genetic diversity values observed in the fish farms (measured as A_r_, H_obs_, and H_exp_ for SSR and as MAF, H_obs_, and H_exp_ for SNPs) were not significantly different from the WILD values (*p* > 0.75 for A_r_, H_obs_, and H_exp_, Kruskall–Wallis tests for SSRs; *p* > 0.90 for MAF, H_obs_, and H_exp_, Kruskall–Wallis tests for SNPs), which showed A_r_ = 3.420 ± 1.0005, H_obs_ = 0.441 ± 0.213, and H_exp_ = 0.434 ± 0.214 for SSRs and MAF = 0.275 ± 0.132, H_obs_ = 0.385 ± 0.171, and H_exp_ = 0.370 ± 0.130 for SNPs (Table 1).

All SNP loci were in accordance with HWE after Bonferroni correction (*p* = 0.001), even when populations were subjected to global tests for all loci. By contrast, some SSR loci departed from HWE after Bonferroni correction (*p* = 0.006), such as locus Pm4 (FF2, FF3, and FF6) and locus Pm5 (FF4). Null alleles were detected in the locus Pm4 (FF2, FF3, FF4, and FF6) and locus Pm11 (FF2) (Table S1). Neither scoring errors nor allele drop-out were detected. Four fish farms (FF1, FF2, FF4, and FF6) departed from HWE (*p* < 0.05) when applying global tests for SSRs. However, only FF4 presented significant Hardy–Weinberg disequilibrium after Bonferroni correction (*p* < 0.006).

Overall population *F*_IS_ values varied similarly between SSR (−0.113 and 0.212) and SNP loci (−0.082 to 0.056). However, no significant deviations from zero of *F*_IS_ values were found in the fish farms when Bonferroni correction was applied for SNPs (*p* = 0.001) and SSRs (*p* = 0.006). Additionally, *F*_IS_ values were not considered significantly different from zero over all loci for both markers (*p* > 0.05), presenting low 95% confidence interval values for SSRs (−0.022 to 0.135) and SNPs (−0.075 to 0.011) when bootstrapping over loci analyses were performed.

Except for in FF2 and FF6, SNP markers showed LD between pairs of loci in most fish farms: FF1 (7 pairs), FF7 (5), WILD (4), FF5 (2), FF3 (1), and FF4 (1) (*p* < 0.001). For SSR markers, LD was detected in FF4 (5 pairs), FF2 (2), FF1 (1), FF5 (1), and WILD (1) (*p* < 0.01). However, in both markers, the observed LD was not homogeneous among populations or loci (Table S3).

Reduced effective population size was detected in fish farms when compared to WILD for both SNP and SSR sets (except for FF6 in SSR analysis). N_e_ estimation showed values ranging from 2.3 in FF1 to 20.2 in WILD using SNP markers. In relation to SSRs, N_e_ values ranged from 7.0 in FF4 to 60.8 in FF6 (Table 2). The rate of inbreeding (∆F) was detected by considering N_e_ estimates and resulted in the lowest values in FF6 for SSRs (0.01) and in WILD for SNPs (0.02) and the highest values in FF4 for SSRs (0.07) and in FF1 for SNPs (0.22). Evidence of recent reductions in population size was found in all fish farms (M-ratio < 0.68) by considering SSR analysis (Table 2) characterizing bottleneck events.

**Figure 1:**
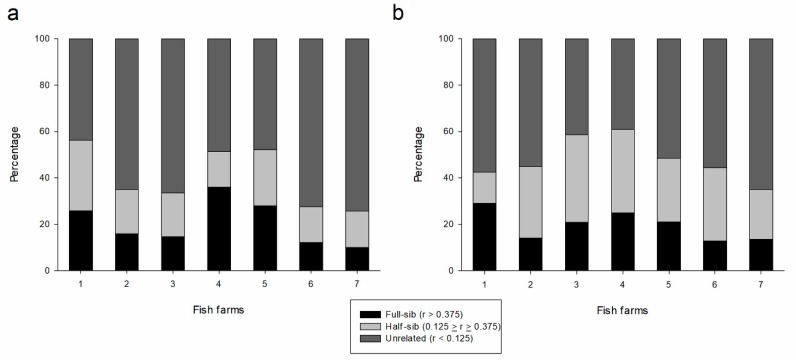
Distribution of kinship values (r_xy_) by Wang’s estimator of fish farms (FF1–FF7) in SSR (**a**) and SNP (**b**) datasets. Threshold values for kinship analysis: unrelated (r < 0.125), half sibling (0.125 ≤ r ≤ 0.375), and full sibling (r > 0.375) individuals.

After the detection of reduced effective population sizes, kinship evaluation showed that most of the fish farms had related individuals (full sibling and half-sibling) in a proportion of at least 25%, as estimated from both marker sets (Table 3; Figure 1), with significant probability of mating between related breeders. The percentage of related individuals was notable in FF4 (61.0% and 51.4% for SNPs and SSRs, respectively) with a high proportion of full sibling individuals (25.0% and 36.0% for SNPs and SSRs, respectively). Although FF7 showed a considerable proportion of related individuals, this farm showed the highest rate of unrelated individuals (65.1% and 74.3% for SNPs and SSRs, respectively) (Figure 1). Confidence intervals (95%) of pairwise relatedness for each dyad are presented in Table S4. The PE2 for each locus for SSRs ranged from 0.04 (locus Pm3) to 0.31 (loci Pm2 and Pm7) with an exclusion probability of 0.81 for all loci. Considering SNPs, PE2 values ranged from 0.01 (loci 41_428, 437_455, 458_2209, and 83_761) to 0.12 (loci 1013_445, 213_629, 260_818, 391_875, 470_159, and 4_231) with an exclusion probability of 0.07 for all loci (Table S5).

Global *F*_ST_ values suggested low genetic differentiation among populations when using SSRs (*F*_ST_ = 0.080, *p* < 0.006) or SNPs (*F*_ST_ = 0.067, *p* < 0.001). Pairwise *F*_ST_ values were also calculated for fish farms and significant differentiation was found between most pairs when considering SNP and SSR sets (*p* < 0.001 and *p* < 0.006, respectively) (Table 4). Low to high genetic differentiation was observed among the population pairs. Pairwise *F*_ST_ values for SSR loci were mostly significant (*p* < 0.006) and ranged between −0.002 and 0.204. The highest significant *F*_ST_ values (*p* < 0.006) were observed between WILD and FF4 (*F*_ST_ = 0.204) and FF2 and FF4 (*F*_ST_ = 0.143). Conversely, the lowest genetic differentiation was detected between FF3 and FF6 (*F*_ST_ = −0.002, *p* > 0.05). Regarding SNPs, the highest genetic differentiation (*p* < 0.001) was between FF2 and FF4 (*F*_ST_ = 0.146). Conversely, WILD and FF7 (*F*_ST_ = 0.032) registered the lowest genetic differentiation (*p* < 0.001).

**Figure 2:**
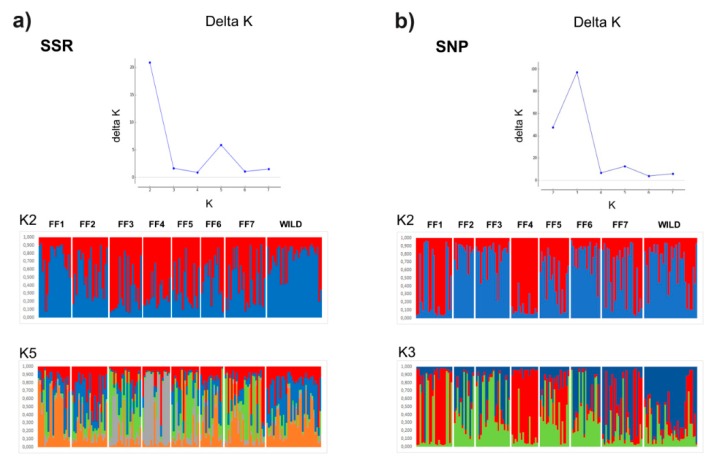
ΔK statistics in STRUCTURE analysis as a function of the number of putative genetic clusters (K) for pacu populations. Coefficient of ancestry of pacu individuals collected from seven cultivated stocks (FF1–FF7) and one wild population (WILD), based on 8 SSR (**a**) and 32 SNP loci (**b**). Each vertical bar represents an individual. The fish farms are separated by vertical white lines. The color proportions of each bar correspond to individuals’ estimated membership fractions of each of the clusters.

To evaluate the level of admixture among samples, Bayesian model-based clustering analyses were performed based on the ΔK distribution by examining SSR and SNP loci. The selection of the estimated number of clusters in the dataset was based on the number of analyzed fish farms (K = 1 to 8). According to the analysis based on the Evanno method [30], the hypothesis of occurrence of K = 1 was discarded due to the higher −ln P(K) values found in all analyses (data not shown). The results showed that K values of 2 and 3 for SSRs and SNPs, respectively, were the most suitable to explain the population structure of pacu stocks (Figure 2). For the SNP dataset in K = 3, moderate clustering was found between fish farms, with three putative clusters composed of (1) FF1 and FF4; (2) FF2, FF3, and FF6; and (3) FF7 and WILD. In addition, FF5 seems to be represented as an admixture between the presented clusters (Figure 2a). However, considering the SSR analysis in K = 2, two genetic clusters were detected: (1) FF1, FF2, and WILD and (2) FF3, FF4, and FF5. The remaining fish farms (FF6 and FF7) were considered an admixture of both genetic groups (Figure 2b). Moreover, the analysis showed a structure for K = 5 in SSRs in which a clear cluster is composed of FF4. Hence, these analyses confirmed the estimated results of pairwise *F*_ST_ analyses.

Despite the genetic structure among populations being confirmed by STRUCTURE analysis, AMOVA analysis based on groups supported by the results of STRUCTURE showed that the higher percentage of genetic variation was assigned to differentiation within populations: 92.4% by SNPs (*p* < 0.001) and 91.1% by SSRs (*p* < 0.0001). The estimated variation between groups (*F*_CT_) was only 2.2% (*p* = 0.01) for SNPs and 3.9% (*p* = 0.02) for SSRs. In addition, the variation among populations and within groups (*F*_SC_) presented 5.3% of the genetic variation (*p* < 0.001) for SNPs and 5.0% (*p* < 0.0001) for SSRs.

## 4. Discussion

Currently, the methodology for breeding programs is well established in model species of worldwide aquaculture [37]. On the other hand, there are few studies applying genetic markers for the development of genetic selection programs in Neotropical fish. Several practices associated with Neotropical fish production, especially those related to the management of broodstock, may reduce the effective population size [38]. These practices are generally linked to the lack of registration and control of the broodstock, such as information on its origin, kinship, and mating record, and maintenance of the same stock over several generations, which results in increased susceptibility to inbreeding depression that compromises the foundation of hatchery stocks when starting breeding programs [39,40]. Therefore, our results demonstrate the importance of this study, which can be considered the baseline to create the basal broodstock for initial breeding programs of pacu, one of the most important native species of South American aquaculture.

Considering SSR loci, the analysis of genetic diversity parameters estimated on pacu broodstocks showed low values for the number of alleles (ranging around 4) and heterozygosity (ranging around 0.500). It was expected that cultivated stocks would present low genetic diversity values, as herein observed; these low values characterize populations with genetic drift events due to low effective population sizes and, consequently, recent bottleneck effects/founder events, similar to other studies in a related Neotropical species, *Piaractus brachypomus* [41]. However, the low levels of genetic diversity in farmed stocks were also shared by the WILD reference population. These values show lower results when compared to previous studies of genetic diversity in pacu natural populations based on SSRs [17] that reported a higher mean number of alleles (8.5 alleles) and higher heterozygosity values (H_exp_ ~ 0.600). Therefore, our hypothesis is that the WILD population may be threatened due to the high level of exploitation imposed by commercial and/or recreational fishing, since the region where the WILD population was collected is a famous fishing spot in Brazil [42]. Moreover, the Paraná River has been drastically impacted by dam barriers and pollution effects [17,42] which could negatively affect natural populations of pacu.

Although there is no comparative research of the genetic diversity of pacu populations using SNP markers, the values of heterozygosity (i.e., H_obs_ and H_exp_) and MAF indicated low genetic variability in the farmed populations studied. This hypothesis was based on similar values found in WILD and sampled fish farms, considering that wild populations of pacu have no expressive heterozygosity values when using microsatellite loci. In addition, the diversity values herein observed were similar to those in other fish studies involving SNP analyses [43,44,45].

In general, farmed stocks have the tendency to show reduced genetic variability over generations due to artificial selection and a reduced number of breeders in the initial base population [11,46,47,48]. Therefore, it is important to evaluate how the genetic diversity can be maintained when considering the possibility of bottleneck events due to the low effective population size shown in fish farms. The significant bottlenecks detected for all stocks and their low N_e_ values (except for WILD) must receive special attention, since our results will serve to find a base population of pacu for upcoming breeding programs.

Kinship analysis has been an essential tool in genetic pre-breeding programs of fish to reduce inbreeding rates by directing the mating of unrelated individuals [49,50,51,52]. Except for FF7, which showed a high proportion of unrelated individuals for both markers (65.1% by SNPs and 74.3% by SSRs), all fish farms showed a substantial number of related individuals (half sibling or full sibling); this outcome results in a higher inbreeding risk, which can affect morphological and viability traits [10]. Thus, molecular identification of individuals is necessary to effectively monitor the genetic variability of the stocks and to assess how this variation can be maintained through selective mating [13,27]; this is especially the case in FF4, which presented a high proportion of pairwise kinship, the lowest value of effective population size, and a higher rate of inbreeding.

Impacts related to insufficient individuals used in hatchery productions and their interference with the genetic diversity of cultivated populations have been studied for important species cultivated worldwide, such as Atlantic salmon [53]. Similar studies in Neotropical species are fundamental and indispensable for ensuring the correct functioning of initial breeding programs, mainly due to the traditional practices related to the management of Neotropical broodstocks and the lack of genetic information of this species with high potential for production.

In the present study, our initial hypothesis was based on the fact that pacu farmed stocks did not have gene flow because the broodstocks are geographically isolated and producers frequently do not perform exchange of breeders among the fish farms. Therefore, we would expect higher values of *F*_ST_ estimates indicating genetic differentiation (driven by genetic drift and isolation), as detected in FF4, similar to in previous studies carried out in other related species such as *P. brachypomus* [41]. However, overall *F*_ST_ values suggested significant genetic differentiation of 0.067 by SNPs and 0.080 by SSRs (both values with *p* < 0.05), indicating low differentiation between the farmed stocks. This may be explained using two hypotheses: (i) stock foundation based on sharing of breeders among fish farms, resulting in genetic similarities between broodstocks, and/or (ii) stock foundation in the fish farms based on the capture of wild breeders, which are characterized as belonging to a panmictic unit due to the lack of genetic structure in natural populations [15,17], particularly because pacu have high gene flow capacity due to their migratory behavior in the wild.

The STRUCTURE analysis revealed differences between SNPs and SSRs, showing three and two clusters for the pacu stocks, respectively. In this study, the dataset of SNPs originated from the pacu liver transcriptome and these SNPs were annotated mostly in genes related to productive traits, including SNPs classified as non-synonymous mutations [18]. Therefore, both markers might differ in their ability to detect population structure; SNPs are mainly gene-associated while SSRs are expected to be neutral markers, which results in different mutation rates. Neutral markers, such as SSRs, are widely used to perform genetic variation analysis mainly in natural populations, while gene-associated markers could be more useful to analyze the variability of organisms in response to artificial selection [54,55]. This can explain the differentiation of FF1/FF4 in relation to the other fish farms as revealed by SNPs, particularly due to the breeding management practices carried out in these fish farms, such as mass selection to obtain individuals with better growth performance. To design suitable breeding programs in terms of genetic diversity in farmed pacu stocks, we also assume that it is better to use the information of the clusters generated by the SNPs. However, as SNPs are generally biallellic and with lower polymorphism compared to SSRs, additional SNPs markers need to be included in further analysis to achieve better conclusions about the genetic structure of pacu farmed stocks.

The results of this study are aimed to provide initial knowledge about the genetic profile of pacu stocks in different fish farms, considering the importance of pacu to South America aquaculture and the necessity to offer subsidies for the development of its production. The results provide information relevant to one of the most important cultivated Neotropical species. The genetic variability and differentiation of stocks and fish farm profiles considering the risks of inbreeding and the necessity of directed matings of the stocks should be known in order to take appropriate actions for the creation of the base population. In conclusion, the SNP and SSR sets showed their applicability in a pre-breeding program, particularly in delineating the formation of the best families in terms of genetic variability and genetic structure.

## Figures and Tables

**Table 1:** Mean of diversity parameters per population in pacu.

Samples	SSR							SNP					
	N	Na	Ar	H_obs_	H_exp_	HW	*F*_IS_	N	MAF	H_obs_	H_exp_	HW	*F*_IS_
WILD	34	4.00 ± 1.31	3.42 ± 1.01	0.441 ± 0.213	0.434 ± 0.214	0.706	–0.018	34	0.275 ± 0.132	0.385 ± 0.171	0.370 ± 0.130	0.293	0.056
FF1	20	3.50 ± 0.93	3.33 ± 0.86	0.581 ± 0.217	0.524 ± 0.155	0.009	–0.113	23	0.285 ± 0.133	0.394 ± 0.194	0.382 ± 0.131	0.060	−0.064
FF2	22	3.88 ± 1.55	3.62 ± 1.35	0.460 ± 0.254	0.534 ± 0.208	0.006	0.140	13	0.328 ± 0.114	0.431 ± 0.169	0.434 ± 0.101	0.892	−0.034
FF3	20	3.88 ± 1.36	3.66 ± 1.27	0.500 ± 0.171	0.556 ± 0.125	0.325	0.104	22	0.269 ± 0.138	0.382 ± 0.182	0.363 ± 0.146	0.998	−0.025
FF4	17	4.00 ± 1.07	3.81 ± 1.02	0.596 ± 0.315	0.552 ± 0.204	**0.000**	–0.081	17	0.297 ± 0.136	0.425 ± 0.186	0.394 ± 0.127	0.787	−0.040
FF5	17	3.50 ± 1.07	3.36 ± 1.02	0.471 ± 0.206	0.479 ± 0.181	0.751	0.018	19	0.270 ± 0.150	0.370 ± 0.174	0.361 ± 0.145	0.983	0.008
FF6	14	3.50 ± 0.75	3.50 ± 0.76	0.438 ± 0.200	0.551 ± 0.112	0.013	0.212	19	0.273 ± 0.127	0.398 ± 0176	0.376 ± 0.130	0.988	−0.062
FF7	25	4.50 ± 1.20	3.97 ± 1.19	0.530 ± 0.177	0.572 ± 0.170	0.169	0.074	26	0.270 ± 0.144	0.342 ± 0.148	0.362 ± 0.147	0.947	−0.082

Legend: WILD, Paraná river population; FF1–FF7, farm fish stocks; N, number of individuals; N_a_, number of alleles; A_r_, allelic richness; H_obs_, observed heterozygosity; H_exp_, expected heterozygosity; HW, overall loci *p* values of Hardy-Weinberg equilibrium; *F*_IS_, inbreeding coefficient per population; MAF, minimum allele frequency. Significant value of Hardy-Weinberg disequilibrium in simple sequence repeats (SSRs) after Bonferroni correction (*p* < 0.006) is in bold. There are not significant values of heterozygote (negative values of *F*_IS_) and homozygote excess (positive values of *F*_IS_) for SRRs (*p* > 0.006) and single-nucleotide polymorphisms (SNPs) (*p* > 0.001) after Bonferroni correction.

**Table 2:** Bottleneck tests by M-ratio test. Effective population size (N_e_) parameter based on heterozygosity excess and inbreeding coefficient (∆F = 1/2N_e_).

Samples	SSR			SNP	
	M-ratio	N_e_	∆F	N_e_	∆F
WILD	0.31 ± 0.14	32.0 (2.8−inf.)	0.02	20.2 (12.7–35.2)	0.02
FF1	0.31 ± 0.16	18.1 (18.1–inf.)	0.03	2.3 (1.8–2.9)	0.22
FF2	0.31 ± 0.16	7.8 (7.8–inf.)	0.06	3.2 (2.0–10.7)	0.16
FF3	0.30 ± 0.15	10.0 (3.3–28.9)	0.05	8.7 (4.6–15.8)	0.06
FF4	0.33 ± 0.17	7.0 (2.5–20.9)	0.07	4.3 (2.5–8.9)	0.12
FF5	0.41 ± 0.30	21.1 (2.9–inf.)	0.02	12.5 (6.5–28.9)	0.04
FF6	0.38 ± 0.28	60.8 (5.3–inf.)	0.01	9.9 (5.7–17.8)	0.05
FF7	0.30 ± 0.14	16.1 (5.8–86)	0.03	4.4 (2.7–7.5)	0.11

Confidence interval used in analysis = 95%.

**Table 3:** Kinship analysis for the 8 SSR and 32 SNP loci in farmed populations of pacu (*Piaractus mesopotamicus*) (FF1–FF7), according to Wang’s r_xy_ coefficient. Values are represented in percentage.

Fish Farm	SSR			SNP		
	Unrelated	Half-sib	Full-sib	Unrelated	Half-sib	Full-sib
FF1	43.7	30.5	25.8	57.5	13.5	29.0
FF2	64.9	19.0	16.0	55.1	30.8	14.1
FF3	66.3	18.9	14.7	41.5	37.7	20.8
FF4	48.5	15.4	36.0	39.0	36.0	25.0
FF5	47.8	24.3	27.9	51.5	27.5	21.0
FF6	72.5	15.4	12.1	55.6	31.5	12.9
FF7	74.3	15.7	10.0	65.1	21.3	13.6

**Table 4:** Pairwise *F*_ST_ estimates from the 8 SSR loci (below diagonal) and from the 32 SNP loci (above diagonal) in the wild and farmed populations of pacu (*Piaractus mesopotamicus*).

	FF1	FF2	FF3	FF4	FF5	FF6	FF7	WILD
FF1	-	**0.096**	**0.057**	**0.066**	**0.085**	**0.081**	**0.041**	**0.052**
FF2	**0.042**	-	**0.067**	**0.146**	**0.104**	**0.042**	**0.092**	**0.053**
FF3	**0.092**	0.042	-	**0.136**	**0.095**	**0.054**	**0.051**	**0.051**
FF4	**0.130**	**0.143**	**0.090**	-	**0.089**	**0.093**	**0.067**	**0.094**
FF5	**0.111**	**0.063**	0.043	**0.145**	-	**0.068**	**0.063**	**0.070**
FF6	0.027	0.005	–0.002	**0.095**	0.040	-	**0.052**	**0.036**
FF7	**0.035**	**0.046**	**0.063**	**0.115**	**0.055**	0.027	-	**0.032**
WILD	**0.033**	**0.039**	**0.120**	**0.204**	**0.115**	**0.041**	**0.081**	-

Significant *F*_ST_ values (*p* < 0.05) are in bold.

## Supplementary Materials

The following are available online at https://www.mdpi.com/2073-4425/10/9/668/s1, Table S1: Genetic variability parameters of 8 SSR loci in pacu populations, Table S2: Genetic variability parameters of the 32 SNP loci in pacu populations, Table S3: Linkage disequilibrium (LD) between the molecular markers, Table S4: Kinship values (r_xy_) by Wang’s estimator of fish farms (FF1–FF7) in SSR and SNP datasets with confidence intervals (95%) of pairwise relatedness for each dyad, Table S5: Parentage exclusion probabilities of loci for SSR and SNP datasets (PE2).
